# Association of TyG-BMI and malnutrition with reduced survival outcomes in patients with cancer: a prospective study

**DOI:** 10.3389/fnut.2026.1887549

**Published:** 2026-07-30

**Authors:** Yi-Zhen Gong, Bao-Min Zhang, Cun Liao, Li-Chen Zhu, Guo-Tian Ruan

**Affiliations:** 1 Department of Clinical Research, Guangxi Medical University Cancer Hospital, Nanning, Guangxi, China; 2 Department of General Surgery, Qilu Hospital (Qingdao), Cheeloo College of Medicine, Shandong University, Qingdao, China; 3 Department of Colorectal and Anal Surgery, The First Affiliated Hospital of Guangxi Medical University, Nanning, China; 4 Guangxi Key Laboratory of Enhanced Recovery After Surgery for Gastrointestinal Cancer, Nanning, China; 5 Department of General Surgery, Beijing Friendship Hospital, Capital Medical University, Beijing, China

**Keywords:** inflammation, insulin resistance, malnutrition, overall survival, TyG-BMI index

## Abstract

**Background:**

Several triglyceride-glucose (TyG)-related indices are used to assess insulin resistance (IR), but the better indicator for patients with cancer remains undetermined. The prognostic value of TyG-body mass index (TyG-BMI), its association with malnutrition, and underlying mediating mechanisms in large Chinese multicenter cohorts need further clarification.

**Methods:**

This prospective, multicenter study enrolled 5,221 patients with cancer from the INSCOC cohort. We compared the prognostic performance of TyG-BMI with other TyG-related IR indices. Logistic regression was used to explore the relationship between TyG-BMI and malnutrition (PGSGA). Cox regression and mediation analyses were performed to evaluate prognostic value and mediating pathways.

**Results:**

TyG-BMI was identified as a useful indicator TyG-related IR index, with the highest time-dependent AUC values and C-index. High TyG-BMI was independently associated with better overall survival (adjusted HR = 0.83, 95%CI: 0.76–0.91, *p* < 0.001) and lower malnutrition risk (adjusted OR = 0.42, 95%CI: 0.37–0.48, *p* < 0.001). The prognostic benefit was most significant in esophageal and colorectal cancer. Exploratory pathway decomposition analyses showed that PGSGA (48.2%), CRP (13.6%), KPS (18.5%), and ECOG PS (18.9%) accounted for partial components of the correlation between TyG-BMI and overall survival. Low TyG-BMI combined with malnutrition reduced 1-year survival in patients with cancer (1-year survival rate = 70.7%; median survival time = 27.2 months).

**Conclusion:**

TyG-BMI, a simple composite index based on routine clinical indicators, is a TyG-related insulin resistance index for Chinese patients with cancer. High TyG-BMI is significantly associated with a lower risk of malnutrition and better overall survival. Exploratory pathway analyses indicated nutritional status, systemic inflammation and physical function partially contributed to the crude correlation between TyG-BMI and survival. TyG-BMI and malnutrition are jointly associated with reduced survival outcomes in patients with cancer, supporting its clinical value for prognostic stratification and nutritional-metabolic management.

## 1 Introduction

Cancer is a leading cause of mortality worldwide, with increasing incidence and disease burden reported in both developed and developing regions (1, 2). Despite advances in surgical techniques, chemotherapy, radiotherapy, targeted therapy, and immunotherapy, long-term survival of patients with cancer remains unsatisfactory, largely due to tumor recurrence, metastasis, treatment intolerance, and systemic comorbidities. Among these comorbid conditions, metabolic disorders and nutritional deterioration are two of the most prevalent and impactful issues that affect the entire disease course from diagnosis to survival (3, 4).

Insulin resistance (IR) is a core pathological feature of metabolic disturbance in patients with cancer, characterized by impaired insulin-mediated glucose uptake and utilization in peripheral tissues (5, 6). Mounting evidence has confirmed that IR is closely associated with tumor initiation, progression, epithelial-mesenchymal transition, angiogenesis, and treatment resistance (7, 8). Hyperinsulinemia caused by IR can activate the PI3K/Akt/mTOR signaling pathway, promoting tumor cell proliferation and inhibiting apoptosis (6, 9). Meanwhile, IR induces chronic low-grade inflammation, oxidative stress, and lipid metabolism disorders, further accelerating cancer development (10). Traditional IR evaluation indexes such as homeostasis model assessment of insulin resistance (HOMA-IR), quantitative insulin sensitivity check index (QUICKI), and McAuley index require simultaneous detection of fasting insulin and blood glucose, which increases clinical detection costs and is not routinely included in basic laboratory examinations for patients with cancer, limiting their wide application in clinical practice (11–13).

In recent years, simple and convenient IR surrogate indicators based on fasting blood glucose and triglycerides have attracted extensive attention (14, 15). The triglyceride-glucose index (TyG), calculated by fasting triglycerides and blood glucose, has been widely verified in metabolic diseases such as type 2 diabetes, metabolic-associated fatty liver disease, and hypertension as a reliable and stable marker of IR (11–15). Compared with HOMA-IR, TyG has the advantages of easy access to indicators, simple calculation, and good reproducibility (16, 17). Further studies found that combining TyG with body mass index (BMI) to construct the composite index TyG-BMI can better reflect the interaction between IR, body fat distribution, and nutritional status, and has higher predictive value for metabolic diseases and tumors than a single index (18–22).

Malnutrition is another common complication in patients with cancer, with a prevalence rate of 40–80% reported in different tumor types (23). Malnutrition not only leads to weight loss, muscle atrophy, and decreased immune function but also increases the risk of postoperative complications, chemotherapy-related adverse reactions, and hospital stays, seriously reducing quality of life and shortening survival time (24, 25). The Patient-Generated Subjective Global Assessment (PGSGA) is a dedicated nutritional risk assessment tool recommended by the European Society for Clinical Nutrition and Metabolism (ESPEN) and the American Society for Parenteral and Enteral Nutrition (ASPEN) for patients with cancer, which can comprehensively evaluate nutritional intake, physical condition, and disease impact (26).

In recent years, studies have explored the relationship between TyG, TyG-BMI and the prognosis of single tumors such as lung cancer, gastric cancer, colorectal cancer, and nasopharyngeal carcinoma (27–30). However, most of these studies focus on the predictive value of TyG-BMI for survival, and few pay attention to its correlation with malnutrition in patients with cancer. In addition, there is still a lack of large-sample pan-cancer research to confirm the stability and universality of TyG-BMI as a prognostic marker, and the potential correlational pathways linking TyG-BMI to survival outcomes remain unclear.

Therefore, in this large Chinese multi-center cohort of patients with cancer, we aimed to identify the useful triglyceride-related IR index, evaluate the prognostic potential of TyG-BMI, explore its association with nutritional status, and clarify the underlying mediating factors. We hypothesized that TyG-BMI is independently associated with overall survival and malnutrition, and could serve as a simple and practical biomarker for survival stratification and nutritional-metabolic management in patients with cancer.

## 2 Materials and methods

### 2.1 Study population

This study was based on the Investigation on Nutrition Status and its Clinical Outcome of Common Cancers (INSCOC) study (Registration number: ChiCTR1800020329). The INSCOC study is a prospective, multi-center cohort study enrolling patients with cancer from multiple medical centers in China between July 2013 and December 2021 (31). Eligible participants were aged≥18 years, with pathologically confirmed malignant tumors, and had independent consciousness and normal communication ability. No strict exclusion criteria were applied in this cohort. After excluding missing data, a total of 5,221 cancer patients were finally included in this study (Supplementary Figure S1). Among the 5,221 enrolled patients, a total of 3,072 all-cause deaths were recorded, and the remaining 2,149 participants were censored, corresponding to an overall censoring proportion of 41.16%.

The study protocol was approved by the Ethics Committees of all participating hospitals, and the study was conducted in accordance with the Declaration of Helsinki. Written informed consent was obtained from all participants before enrollment.

### 2.2 Data collection

Data were collected via a standardized case report form by well-trained investigators. Baseline information included demographic characteristics (age, sex), lifestyle factors (smoking status, alcohol consumption), and medical history (diabetes, hypertension, coronary heart disease, and other comorbidities). Clinical and tumor-related data consisted of cancer type, TNM stage, surgical treatment, chemotherapy, radiotherapy, and family history of malignant tumors.

Fasting venous blood samples were obtained after an overnight fast of at least 8 h. Nutritional status was evaluated using the Patient-Generated Subjective Global Assessment (PGSGA). Functional status was assessed using the Karnofsky Performance Status (KPS) and Eastern Cooperative Oncology Group Performance Status (ECOG PS). Information regarding nutritional intervention, length of hospital stays, and hospitalization costs was also extracted from electronic medical records. All data were double-entered and verified to ensure accuracy and completeness.

### 2.3 Calculation of indices

TyG = ln[fasting triglycerides(TG) (mg/dL) × fasting glucose (mg/dL)/2] (32–34).

BMI = weight / height ^2(kg/m^2) (35, 36).

TyG-BMI = TyG × BMI (27–30).

METS-IR and TG/HDL-c were computed as previously described (16, 18, 37).

TG/HDL-c = TG (mmol/L)/ HDL-c(mmol/L).

METS-IR = ln[(2 × fasting glucose(mg/dL) + TG(mg/dL) × BMI(kg/m2]/ln [HDL-c(mg/dL)].

1 mmol/L TG = 88.54 mg/dL TG; 1 mmol/L Blood glucose = 18 mg/dL Blood glucose; 1 mmol/L HDL-c = 38.67 mg/dL HDL-c.

### 2.4 Malnutrition assessment

Nutritional status was evaluated using the validated PGSGA tool (26). Malnutrition was defined based on standard PGSGA scoring criteria.

### 2.5 Study outcomes

The primary endpoint was overall survival OS, defined as the interval from admission to death from any cause or last follow-up. We also observed the association between TyG-BMI and nutritional status assessed by PGSGA.

### 2.6 Statistical analysis

Continuous variables were expressed as mean ± standard deviation (SD) or median (interquartile range, IQR) and compared using t-tests or Mann–Whitney U tests. Categorical variables were presented as counts (percentages) and analyzed using *χ*^2^ tests. In the present study, we determined the cutoff value of TyG-BMI in patients with cancer using the maximum selection rank statistics in the training cohort, which was 202.48 (Supplementary Figure S2). Logistic regression was used to estimate odds ratios (OR) and 95% confidence intervals (CI) for malnutrition. Cox proportional hazard regression was performed to calculate hazard ratios (HR) for OS. Model 1 was unadjusted; Model 2 adjusted for age and sex; Model 3 adjusted for age, sex, tumor stage, tumor type, surgery, chemotherapy, radiotherapy, smoking, alcohol consumption, tumor family history, nutritional intervention, diabetes, hypertension, and coronary heart disease. In the survival analysis of different tumor subgroups, our adjustment variables were Model 4: age, sex, tumor stage, surgery, chemotherapy, radiotherapy, smoking status, alcohol consumption, Family history of tumors, nutrition intervention, diabetes, hypertension, and coronary heart disease. The C-index was used to compare predictive accuracy. Mediation analysis was conducted to assess indirect effects. We performed a sensitivity analysis by excluding patients who died within 6 months in multivariate survival analysis to rule out reverse causality. Exploratory pathway decomposition analyses were performed using the mediation package in R. For each potential mediator, sequential regression models were constructed to decompose the total effect into average causal mediation effect (ACME) and average direct effect (ADE). Bootstrap resampling with 1,000 iterations was utilized to generate 95% confidence intervals for effect estimates. All models were adjusted for clinically relevant confounding covariates. The interaction term between exposure and mediator was not incorporated in the primary analytical model.

Maximally selected rank statistics were applied to calculate the optimal cutoff value of TyG-BMI based on the entire overall cohort. Subsequently, stratified random sampling was adopted to divide the total population into a 70% training subset and a 30% independent internal validation subset. The predictive discrimination performance of the TyG-BMI cutoff determined from the full cohort was further validated in the separate internal validation set, including C-index and time-dependent AUC evaluation.

A two-sided *p* < 0.05 was considered statistically significant. All analyses were performed using R 4.1.1.

## 3 Results

### 3.1 Baseline characteristics

**Figure 1:**
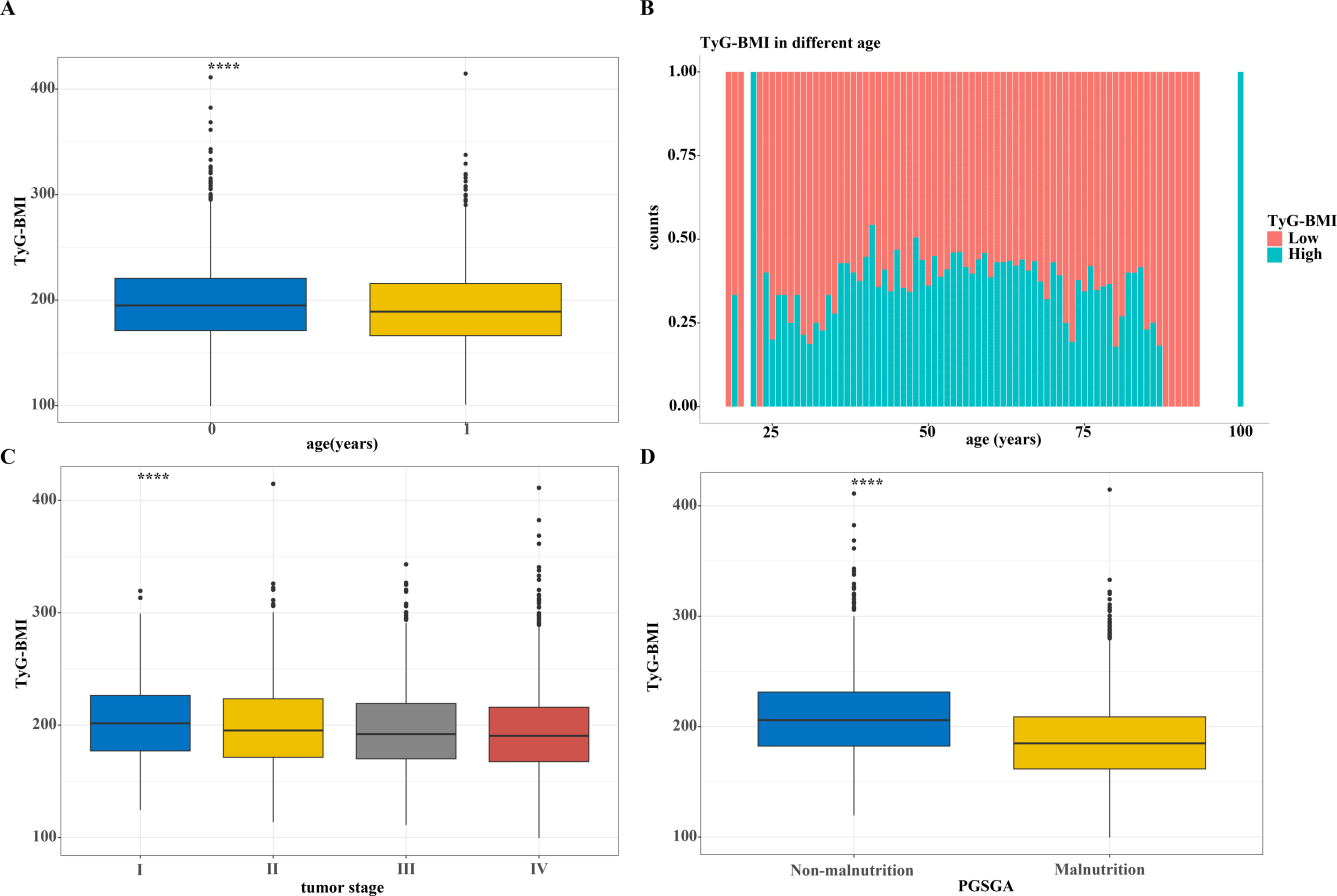
Distribution of TyG-BMI according to age, tumor stage, and PGSGA. **(A)** TyG-BMI levels in patients aged <65 and ≥65 years; **(B)** Overall age distribution of TyG-BMI; **(C)** TyG-BMI levels across different TNM stages; **(D)** TyG-BMI levels between malnutrition and non-malnutrition groups defined by PGSGA. Notes: TyG: triglyceride-glucose index; BMI: body mass index; PGSGA, Patient Generated Subjective Global Assessment.

Among 5,221 included patients, 3,136 were categorized into the low TyG-BMI group and 2085 into the high TyG-BMI group. Patients with high TyG-BMI had higher BMI, triglycerides, fasting glucose, KPS score, and lower CRP, PGSGA score (all *p* < 0.001). They also had higher rates of chemotherapy, and lower rates of malnutrition and nutritional intervention (all p < 0.001; Table 1; Figure 1). The follow-up cutoff date for this cohort was 2021. The median follow-up time was 31.5 months. During the follow-up period, a total of 3,072 all-cause death events occurred. The number of death events across TyG-BMI subgroups was as follows: 1997 in the low group and 1,075 in the high group. Schoenfeld residual tests confirmed that the proportional-hazards assumption held for all Cox models, indicating that Cox proportional hazards regression was suitable for this dataset. Compared with excluded patients, individuals included in the final analytical cohort presented significantly different baseline clinical profiles, including higher disease severity and more comorbid inflammatory conditions, which was consistent with the selective clinical testing strategy for CRP. Detailed comparative results are shown in Supplementary Table S1.

**Table 1:** Baseline characteristics of the study population.

Characteristics	All patients	Low TyG-BMI	High TyG-BMI	*p*-value
	(*n* = 5,221)	(*n* = 3,136)	(*n* = 2085)	
Age, years [mean (SD)]	59.41 (11.15)	59.64 (11.64)	59.07 (10.36)	00.068
Gender (%)				<0.001
Male	3,061 (58.6)	1925 (61.4)	1,136 (54.5)	
Female	2,160 (41.4)	1,211 (38.6)	949 (45.5)	
BMI, kg/m^2^ [mean (SD)]	22.57 (3.51)	20.45 (2.19)	25.77 (2.58)	<0.001
Smoking, yes (%)	2,431 (46.6)	1,524 (48.6)	907 (43.5)	<0.001
Alcohol, yes (%)	1,167 (22.4)	738 (23.5)	429 (20.6)	0.001
Diabetes, yes (%)	530 (10.2)	210 (6.7)	320 (15.3)	<0.001
Hypertension, yes (%)	1,085 (20.8)	484 (15.4)	601 (28.8)	<0.001
Coronary heart disease, yes (%)	268 (5.1)	135 (4.3)	133 (6.4)	0.001
Family history of tumors (%)	871 (16.7)	492 (15.7)	379 (18.2)	0.020
Tumor stage (%)				<0.001
I	441 (8.4)	225 (7.2)	216 (10.4)	
II	943 (18.1)	538 (17.2)	405 (19.4)	
III	1,413 (27.1)	853 (27.2)	560 (26.9)	
IV	2,424 (46.4)	1,520 (48.5)	904 (43.4)	
Surgery, yes (%)	2,596 (49.7)	1,538 (49.0)	1,058 (50.7)	0.240
Radiotherapy, yes (%)	581 (11.1)	332 (10.6)	249 (11.9)	0.139
Chemotherapy, yes (%)	3,312 (63.4)	1924 (61.4)	1,388 (66.6)	<0.001
KPS [mean (SD)]	85.40 (12.35)	84.08 (13.23)	87.38 (10.59)	<0.001
ECOG PS				<0.001
0–1	4,771 (91.4)	2,805 (89.4)	1966 (94.3)	
≥2	450 (8.6)	331 (10.6)	119 (5.7)	
PGSGA [mean (SD)]	6.01 (4.76)	7.04 (5.01)	4.46 (3.86)	<0.001
Nutritional intervention, yes (%)	1,000 (19.2)	695 (22.2)	305 (14.6)	<0.001
Blood glucose, mmol/L [mean (SD)]	5.78 (1.76)	5.44 (1.32)	6.29 (2.18)	<0.001
CRP, mg/L [median (IQR)]	3.71 (13.2)	3.89 (16.56)	3.55(9.44)	<0.001
Triglyceride, mmol/L [mean (SD)]	1.50 (1.49)	1.18 (0.62)	1.98 (2.14)	<0.001
HDL, mmol/L [mean (SD)]	1.22 (0.37)	1.26 (0.41)	1.17 (0.30)	<0.001
TyG [mean (SD)]	8.66 (0.59)	8.42 (0.48)	9.01 (0.56)	<0.001
METS-IR [mean (SD)]	34.60 (6.98)	30.51 (4.53)	40.76 (5.31)	<0.001
TG/HDL [mean (SD)]	3.17 (3.28)	2.46 (2.21)	4.24 (4.20)	<0.001
TyG-BMI [mean (SD)]	196.02 (36.75)	172.12 (19.91)	231.95 (25.20)	<0.001
Length of stay, days [mean (SD)]	13.37 (11.75)	13.58 (11.94)	13.07 (11.47)	0.124
Hospitalization costs, yuan (mean (SD))	25961.98 (40729.67)	26711.86 (42523.82)	24834.09 (37853.46)	0.103

SD, Standard deviation; IQR, Interquartile range; BMI, Body mass index; KPS, Karnofsky performance status; ECOG PS, Eastern cooperative oncology group performance status; PGSGA, Patient-generated subjective global assessment; HDL, High-density lipoprotein; CRP, C-reactive protein; TyG, Triglyceride-glucose index; METS-IR, Metabolic score for insulin resistance.

### 3.2 TyG-BMI: a better IR-related prognostic predictor in patients with cancer

**Figure 2:**
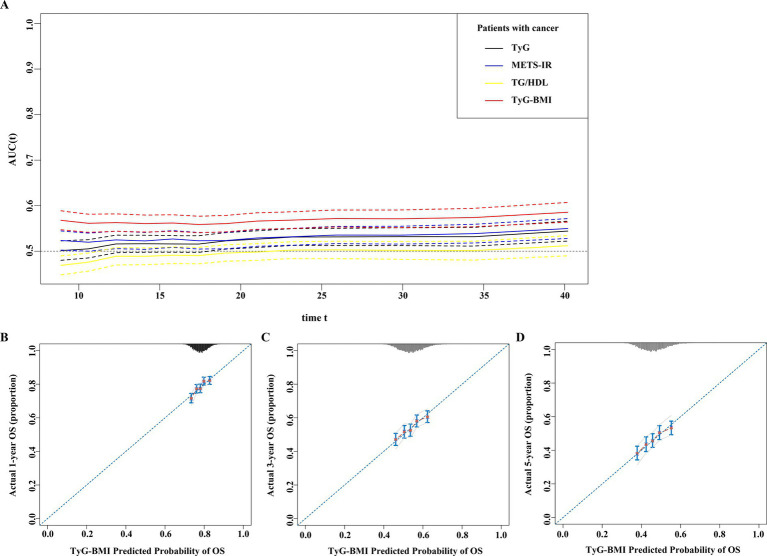
Performance of TyG-BMI for predicting overall survival in patients with cancer. **(A)** Time-dependent AUC curves of TyG-BMI, TyG, METS-IR, and TG/HDL-c for overall survival; **(B-D)** Calibration curves of TyG-BMI for predicting 1-year (B), 3-year **(C)**, and 5-year **(D)** overall survival. Notes: AUC, Area under the curve; TyG: triglyceride-glucose index; BMI: body mass index; METS-IR, metabolic score for insulin resistance; TG, triglyceride; OS, overall survival.

C-index analysis revealed that TyG-BMI had the highest predictive value for OS (0.552, 95% CI: 0.539–0.565) compared with TyG (0.513), METS-IR (0.523), and TG/HDL-c (0.506) (Supplementary Table S2). Time-dependent AUC analysis indicated that TyG-BMI exhibited superior prognostic performance compared with TyG, METS-IR, and TG/HDL-c (Figure 2A). Therefore, TyG-BMI was identified as a better discrimination for survival predictor in patients with cancer.

### 3.3 Comprehensive survival prediction value of TyG-BMI in patients with cancer

#### 3.3.1 Calibration curves

The calibration curves we plotted demonstrated the prognostic value of TyG-BMI for 1-, 3-, and 5-year survival in patients with cancer (Figures 2B–D).

#### 3.3.2 Survival analysis

**Figure 3:**
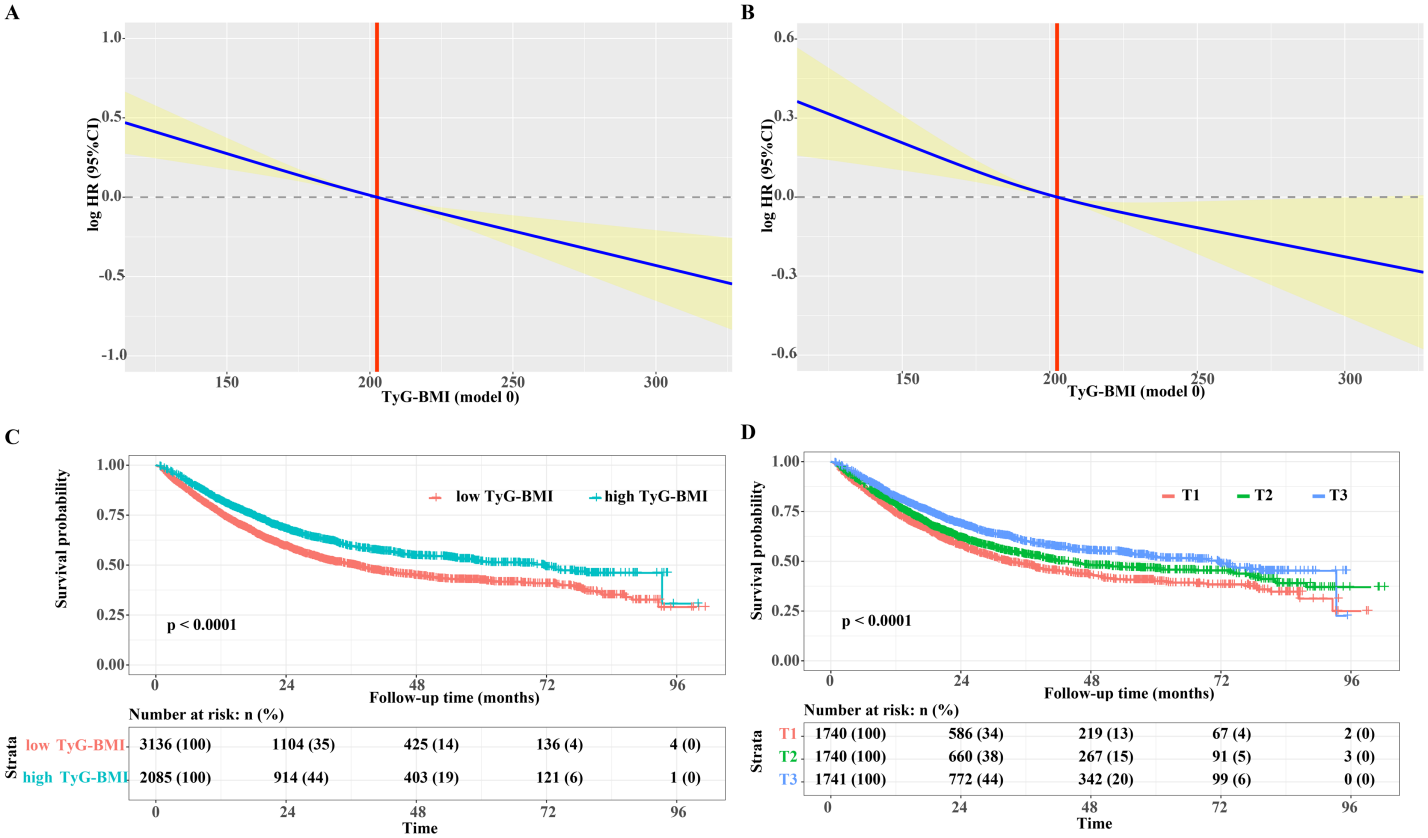
Survival curves of TyG-BMI for overall survival. **(A)** Restricted cubic spline curve showing the association between TyG-BMI and overall survival in unadjusted model; **(B)** Restricted cubic spline curve showing the association between TyG-BMI and overall survival in adjusted model 3; **(C)** Kaplan–Meier survival curves stratified by low and high TyG-BMI groups (cutoff = 202.48); **(D)** Kaplan–Meier survival curves stratified by TyG-BMI tertiles (T1, T2, T3). Notes: TyG: triglyceride-glucose index; BMI: body mass index; HR, hazard ratio.

Kaplan–Meier curves showed significantly better overall survival (OS) in the high TyG-BMI group (*p* < 0.001). Schoenfeld residual tests were applied to assess the proportional-hazards assumption for all Cox regression models. All individual variables and the global test presented *p* > 0.05, indicating no violation of the proportional-hazards assumption and confirming the reliability of our Cox models. In multivariate survival analysis, when TyG-BMI was treated as a continuous variable, each one standard deviation (SD) increase in TyG-BMI was associated with an 11% reduction in the risk of mortality among patients with cancer (*p* < 0.001). In adjusted Cox regression analysis, high TyG-BMI was identified as an independent protective factor (adjusted HR = 0.83, 95% CI: 0.76–0.91, p < 0.001). When TyG-BMI was stratified into tertiles, we observed a decreasing trend in the risk of cancer mortality with ascending TyG-BMI levels (p for trend <0.001; Table 2). Table S3 presents sensitivity analyses restricted to patients surviving at least 6 months after enrollment. Modeling TyG-BMI as a per-SD continuous variable, cutoff-based binary grouping and tertile stratification all revealed that higher TyG-BMI was independently associated with reduced all-cause mortality in both unadjusted and fully adjusted Cox models, except for the non-significant finding of the middle tertile after covariate correction. Significant linear trend associations were also observed across TyG-BMI tertiles (*p* for trend = 0.002), which validated the stable prognostic performance of TyG-BMI (Figures 3).

**Table 2:** Survival analyses.

TyG-BMI	OS (model 1) ^a^		OS (model 2) ^b^		OS (model 3) ^c^	
	Crude HR(95%CI)	Crude *p*	Adjusted HR(95%CI)	Adjusted *p*	Adjusted HR(95%CI)	Adjusted *p*
As continuous (per SD)	0.85 (0.81–0.88)	<0.001	0.86 (0.83–0.90)	<0.001	0.89 (0.85–0.93)	<0.001
By cut-off						
<202.48	ref.		ref.		ref.	
≥202.48	0.73 (0.67–0.80)	<0.001	0.77 (0.70–0.84)	<0.001	0.83 (0.76–0.91)	<0.001
By Tertiles						
T1 (<177.60)	ref.		ref.		ref.	
T2 (177.60–209.27)	0.85 (0.77–0.94)	0.006	0.85 (0.77–0.94)	0.002	0.88 (0.79–0.97)	0.013
T3 (>209.27)	0.69 (0.62–0.76)	<0.001	0.72 (0.65–0.81)	<0.001	0.78 (0.69–0.87)	<0.001
*p* for trend		<0.001		<0.001		<0.001

OS, overall survival; HR, hazard ratio; CI, confidence interval; BMI: body mass index; TyG, triglyceride-glucose index. ^a^Model 1: Unadjusted. ^b^Model 2: Adjusted for age and sex. ^c^Model 3: Adjusted for age, sex, tumor stage, tumor types, surgery, chemotherapy, radiotherapy, smoking status, alcohol consumption, family history of tumors, nutrition intervention, diabetes, hypertension, and coronary heart disease.

#### 3.3.3 Subgroup analysis by tumor type

The survival benefit of high TyG-BMI was significant in gastric cancer (adjusted HR = 0.71, *p* = 0.027), hepatobiliary malignancies (adjusted HR = 0.71, *p* = 0.030), and colorectal cancer (adjusted HR = 0.70, *p* = 0.006), but not in lung, gastric, hepatobiliary, breast, or other tumors (Supplementary Table S4).

### 3.4 Subgroup analysis

**Figure 4:**
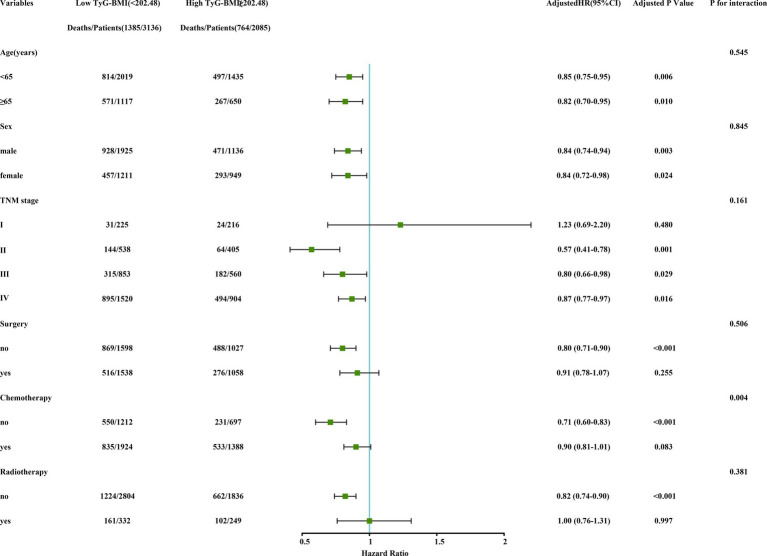
Subgroup analysis of TyG-BMI for overall survival. Forest plot of adjusted hazard ratio for overall survival in the high TyG-BMI group compared with the low TyG-BMI group, stratified by age, sex, TNM stage, surgery, chemotherapy, and radiotherapy. TyG: triglyceride-glucose index; BMI: body mass index; TNM stage, tumor-node-metastasis stage.

In subgroup analysis, we observed that the protective effect was more pronounced in patients undergoing chemotherapy (*p* for interaction = 0.004; Figure 4).

### 3.5 Exploratory pathway decomposition analysis

**Figure 5:**
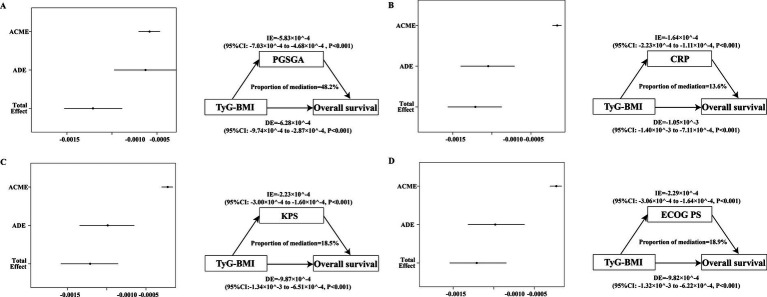
Exploratory pathway decomposition analyses of the association between TyG-BMI and overall survival. **(A)** Correlational pathway contribution of nutritional status (PGSGA); **(B)** Correlational pathway contribution of systemic inflammation (CRP); **(C)** Correlational pathway contribution of physical performance (KPS);**(D)** Correlational pathway contribution of physical performance (ECOG PS). Notes: ACME, average causal mediation effect; ADE, average direct effect; IE, indirect effect; DE, direct effect; TyG: triglyceride-glucose index; BMI: body mass index; CRP: C-reactive protein; KPS, karnofsky performance status; ECOG PS, eastern cooperative oncology group performance status; PGSGA, Patient Generated Subjective Global Assessment.

Exploratory pathway decomposition analyses revealed that PGSGA (48.2%), CRP (13.6%), KPS (18.5%), and ECOG PS (18.9%) accounted for partial components of the association between TyG-BMI and OS, indicating that nutritional status, inflammation, and physical function partially suggested potential correlational associations (Figure 5).

### 3.6 TyG-BMI and malnutrition risk

Logistic regression demonstrated that high TyG-BMI was significantly associated with reduced malnutrition risk. In the unadjusted model, OR = 0.37 (95% CI: 0.33–0.41, *p* < 0.001). After full adjustment for confounders, the association remained robust (adjusted OR = 0.42, 95% CI: 0.37–0.48, *p* < 0.001; Supplementary Table S5).

### 3.7 The relationship between TyG-BMI, PGSGA and 1-year survival in patients with cancer

We further analyzed the impact of TyG-BMI and PGSGA on 1-year survival in patients with cancer. We found that different TyG-BMI levels and nutritional statuses were significantly associated with 1-year survival in these patients. Specifically, among patients with low TyG-BMI, those with malnutrition had a markedly reduced 1-year survival rate (1-year survival rate = 70.7%; median survival time = 27.2 months) (Table 3).

**Table 3:** The relationship between the PGSGA, TyG-BMI and 1-year survival rate in patients with cancer (*n* = 5,221).

Characteristics	Survival parameters	High TyG-BMI	Low TyG-BMI	TyG-BMI	*p* value
		*n* (%)	*n* (%)	*n* (%)	
Non-malnutrition	*n*	1,135	953	2088	<0.001
	Survival rate at 1 year	85.9%	86.1%	86.0%	
	Median survival(month)	NA	82	92.6	
	95% CI	79.2-NA	78.2-NA	79.3-NA	
Malnutrition	*n*	950	2,183	3,133	<0.001
	Survival rate at 1 year	77.3%	70.7%	72.7%	
	Median survival(month)	39.2	27.2	29.8	
	95% CI	33.3–50.4	25.1–30.1	27.4–33.0	
PGSGA	*n*	2085	3,136	5,221	<0.001
	Survival rate at 1 year	82.0%	75.4%	78.1%	
	Median survival(month)	71.3	36.8	44.5	
	95% CI	58.5-NA	32.6–39.6	40.5–51.4	
*p* value		<0.001	<0.001	<0.001	

TyG, triglyceride-glucose index; BMI, body mass index; PGSGA, patient-generated subjective global assessment; 95% CI, confidence interval. Statistical analysis was with *χ*^2^ test for linear-by-linear association, or *χ*^2^ test for 2 by 2 tables.

### 3.8 Internal validation set analysis

The internal validation set consisting of randomly selected 30% participants was used to test the prognostic predictive value of TyG-BMI. The Supplementary Table S6 presents the C-index values of four insulin resistance indicators in the internal validation set. The C-index of TyG-BMI reached 0.557 (95% CI: 0.533–0.581), which was significantly higher than that of TyG (C-index = 0.517, *p* < 0.001) and TG/HDL-c (C-index = 0.502, *p* < 0.001), and numerically superior to METS-IR (C-index = 0.530, *p* = 0.074). Consistent with the C-index results, time-dependent AUC analysis in the same validation cohort (Supplementary Figure S3A) confirmed TyG-BMI possessed better long-term predictive performance for overall survival. Calibration curves for 1-, 3-, and 5-year OS (Supplementary Figures S3B–S3D) showed favorable matching between predicted survival probability and actual observed survival proportion.

## 4 Discussion

In the present large-scale, prospective, multi-center cohort study derived from the INSCOC database, we comprehensively compared multiple TyG-related IR surrogates and found TyG-BMI displayed relatively stronger correlations with malnutrition an OS among Chinese cancer patients. High TyG-BMI was independently associated with a lower risk of malnutrition and better long-term OS, and exploratory pathway decomposition analyses showed nutritional status, systemic inflammation and physical function partially contributed to the correlation between TyG-BMI and survival in this cohort. To our knowledge, this is the first large pan-cancer study to simultaneously compare multiple IR indices, evaluate the prognostic value of TyG-BMI, explore its relationship with PGSGA-assessed malnutrition, and clarify potential mediating pathways in a Chinese multi-center population (18, 20, 31). Our cohort-based observations suggest TyG-BMI might act as an inexpensive, easily accessible useful laboratory marker to assist nutritional screening and crude survival risk assessment, yet its limited discriminative power restricts its standalone use for precise individual risk stratification.

TyG-BMI is a novel composite indicator that integrates triglyceride-glucose index (TyG) and body mass index (BMI), enabling simultaneous reflection of IR, glucose-lipid metabolism, adiposity, and nutritional reserve (18–22). In our head-to-head comparison, TyG-BMI achieved the highest C-index and time-dependent AUC among four commonly used IR surrogates, including TyG, METS-IR, and TG/HDL-c. This predictive ability may be explained by the fact that TyG-BMI captures both metabolic disturbance and nutritional depletion, which are core determinants of cancer progression and treatment tolerance (3, 4, 15). Single IR indices only reflect one aspect of metabolic disorders, whereas TyG-BMI combines metabolic dysfunction with nutritional status, thereby providing a more comprehensive assessment of the systemic condition of patients with cancer (14, 18, 20). Consistent with previous studies in nasopharyngeal carcinoma, lung cancer, and breast cancer, our results confirm that TyG-BMI outperforms single metabolic markers and exhibits relatively stronger correlational links with cancer survival in this pan-cancer cohort (18, 27–30).

We further demonstrated a robust inverse association between TyG-BMI and malnutrition risk evaluated by PGSGA. Patients with elevated TyG-BMI had significantly lower PGSGA scores and a reduced prevalence of malnutrition, suggesting that TyG-BMI may serve as a metabolic marker for early nutritional risk screening (26). Malnutrition is highly prevalent in patients with cancer and is closely associated with treatment intolerance, postoperative complications, prolonged hospital stay, increased medical costs, and poor survival (5, 6, 23, 25). In our study, the low TyG-BMI group not only had a higher risk of malnutrition but also exhibited higher hospitalization costs, which provides preliminary observational evidence for the potential clinical relevance of TyG-BMI. Mechanistically, we hypothesized that low TyG-BMI typically accompanies insufficient energy intake, muscle wasting, fat loss, and disordered glucose and lipid metabolism, all of which are core features of cancer-associated malnutrition. Therefore, TyG-BMI may help clinicians identify high-risk individuals before obvious malnutrition occurs and enable early nutritional intervention.

Survival analyses confirmed that high TyG-BMI was an independent protective factor for OS in patients with cancer, even after extensive adjustment for demographics, tumor characteristics, treatment modalities, nutritional status, and comorbidities. Survival analysis revealed a gradual reduction in mortality risk with increasing TyG-BMI levels, supporting a linear, protective relationship. Notably, the prognostic value of TyG-BMI was tumor-type specific, with more pronounced benefits observed in digestive system cancer. These gastrointestinal tumors are frequently accompanied by eating difficulties, malabsorption, severe metabolic disturbance, and rapid nutritional deterioration, making patients more susceptible to the effects of metabolic-nutritional disorders (21, 28, 29). In contrast, tumors such as lung cancer and breast cancer are less dependent on gastrointestinal function and nutritional intake, which may explain the non-significant findings. These results suggest that TyG-BMI may be particularly valuable for prognostic evaluation in gastrointestinal malignancies.

Pathway decomposition analyses provided preliminary observational clues for understanding the correlation between TyG-BMI and survival. Nutritional status (PGSGA) was the strongest mediator, accounting for 48.2% of the total effect, indicating that nutritional status made up the largest proportion of the correlational links between TyG-BMI and survival in our cohort. Systemic inflammation (CRP) accounted for 13.6% of the effect, supporting the hypothesis that IR promotes chronic inflammation, which in turn accelerates malnutrition and impairs survival (10). Physical performance status (KPS and ECOG PS) also contributed a share of the overall correlational association, consistent with the view that metabolic health and nutritional status directly influence functional capacity (3, 4). Exploratory mediation analyses suggested potential correlational associations linking TyG-BMI to survival mediated by nutritional status, physical performance and systemic inflammation.

The C-index comparison in Supplementary Table S6 directly indicated that TyG-BMI had stronger discriminative power for long-term survival than conventional insulin resistance markers. Combined with the well-fitted time-dependent AUC and calibration curves in Supplementary Figure S3, these internal validation results collectively verified the reliable predictive performance of TyG-BMI in unseen samples of this multi-center population.

The dual biological pathways mediated by the two components of TyG-BMI jointly explain its paradoxical correlation with overall survival in cancer patients. The TyG index reflects peripheral insulin resistance, which drives persistent low-grade systemic inflammation, accelerates tumor cell proliferation and angiogenesis, and impairs anti-tumor immune responses, all of which are unfavorable to long-term prognosis. However, BMI acts as a straightforward indicator of whole-body nutritional reserve: sufficient adipose tissue and skeletal muscle can buffer the pro-inflammatory metabolic stress induced by insulin resistance, maintain intact immune function, and slow down the onset and progression of irreversible cancer cachexia. Most prior relevant research recruited early-stage patients without obvious weight loss or malnutrition, where the adverse metabolic effect of high TyG or elevated BMI dominates outcomes. By contrast, our cohort is dominated by patients with advanced malignancies with a high risk of cachexia. In this context, the protective nutritional benefit of higher BMI outweighs the harmful inflammatory signal carried by increased TyG, ultimately leading to the finding that elevated TyG-BMI is independently associated with improved overall survival. Multiple translational and clinical studies on tumor nutrition, inflammatory metabolism and cachexia were cited to support this competing balance mechanism.

The present study has several notable strengths. First, the large sample size and prospective multi-center design minimize selection bias and enhance the generalizability of results. Second, we directly compared multiple IR-related indices and confirmed the superiority of TyG-BMI. Third, we explored the relationship between TyG-BMI and malnutrition, a critical but understudied topic. Finally, comprehensive sensitivity analyses confirmed the robustness of our conclusions.

Several limitations should also be acknowledged. First, although this is a multi-center cohort, our population was limited to Chinese patients, and further validation in other ethnicities is needed. Second, only baseline TyG-BMI was measured; dynamic changes during treatment may provide additional prognostic information. Third, residual confounding factors may exist despite multivariable adjustment. Fourth, substantial non-random missing CRP data may lead to selection bias. In most participating hospitals of this multi-center cohort, CRP is not a routine admission laboratory test regardless of medical costs. Clinicians only prescribe CRP examinations selectively for patients with severe illness or suspected systemic inflammation, resulting in missing data closely correlated with disease severity. Supplementary Table S1 presents significant baseline differences between patients with complete CRP records and those excluded due to missing CRP. We attempted multiple imputation and inverse probability weighting, yet these statistical methods could not fully resolve the severe baseline imbalance caused by extensive informative missingness. Meanwhile, removing CRP from all models would eliminate the core inflammatory mediator for our pathway mediation analysis, so relevant sensitivity analyses were not conducted. This limitation should be noted when interpreting all inflammation-related mediation findings. Fifth, all exposure, mediator and survival outcome indicators were collected at the same baseline time point, resulting in ambiguous temporal ordering among variables, which restricts robust causal inference. The mediation analytical framework adopted in this work also has inherent constraints when applied to survival data with censored events. Besides, exposure-mediator interaction terms were not incorporated into the primary mediation models. Therefore, all mediation-related results are merely exploratory correlational observations and cannot be regarded as definitive biological or mechanistic evidence. Sixth, specific cut-off values may require further validation in different cancer types. Seventh, TyG-BMI is a composite product index that oversimplifies the independent metabolic and nutritional information conveyed by TyG and BMI separately and ignores critical component heterogeneity. Identical TyG-BMI values can be generated by completely different combinations of TyG and BMI (such as high TyG with low BMI versus low TyG with moderate BMI), and these subgroups carry substantially different prognostic risks, which means continuous TyG-BMI can only reflect overall population trends rather than support precise individualized risk judgment. Moreover, the high proportion of underweight cachectic patients in our advanced cancer cohort is a key population characteristic driving the positive association between TyG-BMI and survival observed herein, which indicates that our conclusions may not be fully generalized to early-stage cancer patients without malnutrition or cachexia.

## 5 Conclusion

In summary, among multiple TyG-related insulin resistance surrogates, TyG-BMI showed relatively stronger correlations with malnutrition risk and overall survival within this Chinese multicenter cancer cohort. High TyG-BMI is associated with reduced malnutrition, lower inflammation, better physical performance, and improved survival, especially in esophageal and colorectal cancer. Exploratory correlational pathway analyses showed nutritional status, systemic inflammation and physical function partially contributed to the correlation between TyG-BMI and OS. Routine calculation of TyG-BMI from standard laboratory tests may serve as a useful reference to support early nutritional intervention and crude risk evaluation, though its modest discriminative power limits its standalone utility for precise individualized risk stratification.

## Data Availability

The original contributions presented in the study are included in the article/Supplementary material, further inquiries can be directed to the corresponding authors.
