# Supplementary material for: Association of TyG-BMI and malnutrition with reduced survival outcomes in patients with cancer: a prospective study
Source: Front Nutr. 2026 Jul 30;13:1887549. doi: 10.3389/fnut.2026.1887549 (PMC13467991; doi:10.3389/fnut.2026.1887549)
Supplement: Supplementary file 1 [file Table_1.DOCX]

**Table S1** **Baseline characteristics comparison.**

| Characteristics | All patients | Excluded patients | P-value |
| --- | --- | --- | --- |
|  | (n=5221) | (n=17562) |  |
| Gender (%) |  |  | <0.001 |
| Male | 3061 (58.6) | 9535(54.3) |  |
| Female | 2160 (41.4) | 8027(45.7) |  |
| BMI, kg/m^2^ (mean (SD)) | 22.57 (3.51) | 21.96 (3.32) | 0.133 |
| Smoking, yes (%) | 2431 (46.6) | 7047(40.1) | <0.001 |
| Alcohol, yes (%) | 1167 (22.4) | 3196(18.2) | 0.001 |
| Diabetes, yes (%) | 530 (10.2) | 1318(7.5) | <0.001 |
| Hypertension, yes (%) | 1085 (20.8) | 2941(16.7) | <0.001 |
| Coronary heart disease, yes (%) | 268 (5.1) | 596(3.4) | <0.001 |
| Family history of tumors (%) | 871 (16.7) | 2351(13.4) | <0.001 |
| Tumor stage (%) |  |  | <0.001 |
| Ⅰ | 441 (8.4) | 2133(12.1) |  |
| Ⅱ | 943 (18.1) | 4205(23.9) |  |
| Ⅲ | 1413 (27.1) | 4578(26.1) |  |
| Ⅳ | 2424 (46.4) | 6646(37.8) |  |
| Surgery, yes (%) | 2596 (49.7) | 7138(40.6) | <0.001 |
| Radiotherapy, yes (%) | 581 (11.1) | 2264(12.9) | <0.001 |
| Chemotherapy, yes (%) | 3312 (63.4) | 9314(53.0) | <0.001 |
| KPS (mean (SD)) | 85.40 (12.35) | 87.13 (12.68) | <0.001 |
| ECOG PS |  |  | 0.300 |
| 0-1 | 4771 (91.4) | 16123(91.8) |  |
| ≥2 | 450 (8.6) | 1439(8.2) |  |
| PGSGA (mean (SD)) | 6.01 (4.76) | 5.53 (4.73) | <0.001 |
| Nutritional intervention, yes (%) | 1000 (19.2) | 3762(21.4) | 0.139 |

Notes: SD: standard deviation; BMI: body mass index; KPS, karnofsky performance status; ECOG PS, eastern cooperative oncology group performance status; PGSGA: patient-generated subjective global assessment.
