# Supplementary material for: Association of TyG-BMI and malnutrition with reduced survival outcomes in patients with cancer: a prospective study
Source: Front Nutr. 2026 Jul 30;13:1887549. doi: 10.3389/fnut.2026.1887549 (PMC13467991; doi:10.3389/fnut.2026.1887549)
Supplement: Supplementary file 2 [file Table_2.DOCX]

**Table S2 c-indexes**

| IR indexes | c-index | Lower 95%CI | Upper 95%CI | Compared P value |
| --- | --- | --- | --- | --- |
| TyG | 0.513 | 0.500 | 0.527 | <0.001 |
| METS-IR | 0.523 | 0.509 | 0.536 | <0.001 |
| TG/HDL-c | 0.506 | 0.500 | 0.527 | <0.001 |
| TyG-BMI | 0.552 | 0.539 | 0.565 | - |

Notes: IR, insulin resistance; 95%CI, 95% confidence interval; TG: triglyceride; HDL-c: high-density lipoprotein cholesterol; BMI, body mass index; TyG: triglyceride-glucose index; METS-IR, IR: metabolic score for insulin resistance; TG/HDL-c, triglyceride/high density lipoprotein cholesterol ratio; TyG-BMI, TyG*BMI index.
