# Supplementary material for: Association of TyG-BMI and malnutrition with reduced survival outcomes in patients with cancer: a prospective study
Source: Front Nutr. 2026 Jul 30;13:1887549. doi: 10.3389/fnut.2026.1887549 (PMC13467991; doi:10.3389/fnut.2026.1887549)
Supplement: Supplementary file 3 [file Table_3.DOCX]

**Table S3 Sensitivity analysis***

| TyG-BMI | OS(model 1)^a^ | |  | OS(model 3)^b^ | |
| --- | --- | --- | --- | --- | --- |
|  | Crude HR(95%CI) | Crude P |  | Adjusted HR(95%CI) | Adjusted P |
| As continuous (per SD) | 0.86 (0.82-0.91) | <0.001 |  | 0.91 (0.86-0.96) | <0.001 |
| By cut-off |  |  |  |  |  |
| <202.48 | ref. |  |  | ref. |  |
| ≥202.48 | 0.76 (0.69-0.85) | <0.001 |  | 0.87 (0.78-0.96) | 0.001 |
| By Tertiles |  |  |  |  |  |
| T1(<177.60) | ref. |  |  | ref. |  |
| T2(177.60-209.27) | 0.87 (0.77-0.98) | 0.020 |  | 0.90 (0.79-1.01) | 0.079 |
| T3(>209.27) | 0.72 (0.64-0.81) | <0.001 |  | 0.81 (0.71-0.92) | 0.002 |
| p for trend |  | <0.001 |  |  | 0.002 |

Notes: ***Excluded patients who died within 6 months.** OS, overall survival; HR, hazard ratio; CI, confidence interval; BMI: body mass index; TyG, triglyceride-glucose index.

^a^ Model 1: Unadjusted.

^b^ Model 3: Adjusted for age, sex, tumor stage, tumor types, surgery, chemotherapy, radiotherapy, smoking status, alcohol consumption, Family history of tumors, nutrition intervention, diabetes, hypertension, and coronary heart disease.
