# Supplementary material for: Association of TyG-BMI and malnutrition with reduced survival outcomes in patients with cancer: a prospective study
Source: Front Nutr. 2026 Jul 30;13:1887549. doi: 10.3389/fnut.2026.1887549 (PMC13467991; doi:10.3389/fnut.2026.1887549)
Supplement: Supplementary file 4 [file Table_4.DOCX]

**Table S4 Survival analyses in different tumor types.**

| Variables | OS(model 1) | | OS(model 4) | |
| --- | --- | --- | --- | --- |
|  | Crude HR(95%CI) | Crude P | Adjusted HR(95%CI) | Adjusted P |
| **Lung cancer** |  |  |  |  |
| TyG-BMI <202.48 | ref. |  | ref. |  |
| TyG-BMI ≥202.48 | 0.83 (0.73-0.96) | 0.005 | 0.90 (0.78-1.03) | 0.125 |
| **Esophageal cancer** |  |  |  |  |
| TyG-BMI <202.48 | ref. |  | ref. |  |
| TyG-BMI ≥202.48 | 0.85 (0.56-1.29) | 0.451 | 0.73 (0.47-1.14) | 0.162 |
| **Gastric cancer** |  |  |  |  |
| TyG-BMI <202.48 | ref. |  | ref. |  |
| TyG-BMI ≥202.48 | 0.62 (0.47-0.83) | 0.001 | 0.71 (0.53-0.96) | 0.027 |
| **Hepatobiliary Malignancies** |  |  |  |  |
| TyG-BMI <202.48 | ref. |  | ref. |  |
| TyG-BMI ≥202.48 | 0.77 (0.57-1.02) | 0.070 | 0.71 (0.52-0.97) | 0.030 |
| **Colorectal cancer** |  |  |  |  |
| TyG-BMI <202.48 | ref. |  | ref. |  |
| TyG-BMI ≥202.48 | 0.69 (0.54-0.87) | 0.002 | 0.70 (0.54-0.90) | 0.006 |
| **Breast cancer** |  |  |  |  |
| TyG-BMI <202.48 | ref. |  | ref. |  |
| TyG-BMI ≥202.48 | 1.17 (0.68-2.00) | 0.573 | 1.43 (0.82-2.48) | 0.206 |
| **Others** |  |  |  |  |
| TyG-BMI <202.48 | ref. |  | ref. |  |
| TyG-BMI ≥202.48 | 0.99 (0.74-1.33) | 0.957 | 1.08 (0.79-1.48) | 0.618 |

Notes: OS, overall survival; HR, hazard ratio; CI, confidence interval; BMI: body mass index; TyG, triglyceride-glucose index.

^a^ Model 1: Unadjusted.

^b^ Model 4: Adjusted for age, sex, tumor stage, surgery, chemotherapy, radiotherapy, smoking status, alcohol consumption, family history of tumors, nutrition intervention, diabetes, hypertension, and coronary heart disease.
