# Supplementary material for: Association of TyG-BMI and malnutrition with reduced survival outcomes in patients with cancer: a prospective study
Source: Front Nutr. 2026 Jul 30;13:1887549. doi: 10.3389/fnut.2026.1887549 (PMC13467991; doi:10.3389/fnut.2026.1887549)
Supplement: Supplementary file 5 [file Table_5.DOCX]

**Table S5 Logistic regression analysis of TyG-BMI and malnutrition.**

| Variables | Crude OR(95%CI) | Crude *P* | Adjusted OR(95%CI) | Adjusted *P* |
| --- | --- | --- | --- | --- |
| **PGSGA*** | | | | |
| TyG-BMI <202.48 | ref. |  | ref. |  |
| TyG-BMI ≥202.48 | 0.37(0.33-0.41) | <0.001 | 0.42(0.37-0.48) | <0.001 |

Notes: OR, odd ratio; CI, confidence interval; BMI: body mass index; TyG, triglyceride-glucose index; PGSGA, Patient Generated Subjective Global Assessment.

*: Adjusted for model 3: age, sex, tumor stage, tumor types, surgery, chemotherapy, radiotherapy, smoking status, alcohol consumption, family history of tumors, nutrition intervention, diabetes, hypertension, and coronary heart disease.
