# Supplementary material for: Association of TyG-BMI and malnutrition with reduced survival outcomes in patients with cancer: a prospective study
Source: Front Nutr. 2026 Jul 30;13:1887549. doi: 10.3389/fnut.2026.1887549 (PMC13467991; doi:10.3389/fnut.2026.1887549)
Supplement: Supplementary file 7 [file Table_7.DOCX]

**Table S7 Hospitals Included in this Study.**

**Hospital Name**

Anhui Provincial Cancer Hospital

Beijing Cancer Hospital

Foshan First People's Hospital

Fujian Provincial Cancer Hospital

Guangdong Provincial People's Hospital

Guigang People's Hospital, Guangxi Province

Affiliated Cancer Hospital of Guangxi Medical University

The First Affiliated Hospital of Guangxi Medical University

Affiliated Cancer Hospital (The First Affiliated Hospital) of Zunyi Medical University, Guizhou Province

Affiliated Cancer Hospital of Harbin Medical University

The Second Hospital of Hebei Medical University (East Campus)

The Fourth Hospital of Hebei Medical University

Bethune First Hospital of Jilin University

The First Affiliated Hospital of Kunming Medical University

Shanghai Tenth People's Hospital

Beijing Shijitan Hospital, Capital Medical University

West China Hospital, Sichuan University

Cancer Hospital of Tianjin Medical University

Wuhan Tongji Hospital

Xijing Hospital

The First People's Hospital of Kashgar Prefecture, Xinjiang

Xingtai People's Hospital

Yunnan Provincial Cancer Hospital

The Second Affiliated Hospital of Zhejiang University School of Medicine

Zhejiang First Hospital

Zhejiang Provincial People's Hospital

Department of Integrated Traditional Chinese and Western Medicine, Zhejiang Provincial Cancer Hospital

Cancer Hospital, Chinese Academy of Medical Sciences

The First Affiliated Hospital of Sun Yat-sen University

Chongqing Daping Hospital

Chongqing Third People's Hospital

Chongqing Cancer Hospital
